# An Integrative Approach to Needs Assessment and Curriculum Development of the First Public Health Major in Singapore

**DOI:** 10.3389/fpubh.2020.00182

**Published:** 2020-06-23

**Authors:** Raymond Boon Tar Lim, Cecilia Woon Chien Teng, Julian Azfar, Diane Bun, Gina Junna Goh, Jeannette Jen-Mai Lee

**Affiliations:** Saw Swee Hock School of Public Health, National University of Singapore and National University Health System, Singapore City, Singapore

**Keywords:** public health, pedagogy, curriculum, undergraduate, education, major, Singapore

## Abstract

Although public health undergraduate education is increasingly popular in the West, studies describing the needs assessment and curriculum development of public health undergraduate education programs are lacking in the Asia Pacific. The objective of this study was to describe the needs assessment and curriculum development of a second major in public health for undergraduates in the National University of Singapore, the first in Singapore. We used the integrated framework for curriculum development in higher education, which consisted of five stages. In Stage 1, the environment was assessed on the need for a new curriculum. Externally, there was a demand for public health workforce in Singapore based on a review of reports from the Ministry of Health and the job portals. Internally, there was a demand from existing students of the university, along with support from the faculty to offer a new curriculum. There was no university in Singapore offering an undergraduate major program in public health. In Stage 2, competencies to be developed were identified from public health job descriptions using job portals, the needs of public health stakeholders, and competencies listed in the public health curriculum accreditation frameworks such as the Council on Education for Public Health. In Stage 3, based on data triangulation, the curriculum was designed as a second major that is offered to all students of the university from year 2 onward. Students have to complete a total of 12 modules, of which 6 are core and 6 are elective. The capstone module is a 320-h internship module where students will be attached to public health–related agencies, organizations, or non-governmental organizations. Our curriculum is generally aligned with undergraduate public health programs in other established universities in the United States of America, United Kingdom, Australia, and Hong Kong. In Stage 4, various pedagogical strategies were identified for the core modules. We are currently at Stage 5 where implementation, monitoring, and evaluation are still being carried out. We hope that the lessons learnt will serve to inform other universities in the Asia Pacific that are considering implementing such programs and broadening their offerings in public health education.

## Introduction

Public health is the art and science of preventing disease, prolonging life, and promoting health through the organized efforts of the society (1). As the focus is on the entire spectrum of health, and not only disease treatment, a diverse workforce is required (2). There are various work roles, from implementing a national screening program, educating construction workers on health and safety, collaborating with vendors to provide healthier food options, working with policymakers to review the healthcare finance framework, to investigating and controlling an outbreak.

Traditionally, public health training has been offered at the postgraduate level, particularly with the Masters in Public Health (MPH) as the main vehicle (3, 4). However, there has been a rapid growth of graduates from undergraduate programs contributing to the public health workforce globally (5). Public health is thus taking on greater prominence in undergraduate education. This could be due to the efforts and advocacy from various organizations. In 2003, the Institute of Medicine (IOM) recommended that all undergraduates should have access to education in public health as it is an essential part of the training of citizens (6). Supported by the Association of Schools and Programs of Public Health (ASPPH) Framing the Future Task Force, schools of public health along with colleges (e.g., liberal arts colleges without existing schools of public health in the United States of America, USA) then took the lead to roll out various undergraduate public health curricula (6, 7). The Task Force provided actionable guidelines to improve education in public health (8). By 2016, 271 institutions in the USA were offering undergraduate public health curricula (9). Correspondingly, the number of public health degree conferrals had also increased from 1,484 in 2004 to 13,605 in 2017 in the USA (10).

While this rise in undergraduate training in public health has spurred related publications, gaps still exist in at least three areas. First, these studies are predominantly centered on the West, particularly the USA, rather than the Asia Pacific region where majority of the world population resides (11). In addition, several public health issues continue to confront the Asia Pacific ranging from emerging infectious diseases to the obesity and diabetes epidemic and urban air pollution (12). For example, cardiovascular disease was the cause of 9.4 million deaths in the region in 2016 (13). Although public health undergraduate education in the USA has become mainstream (14), it is not clear whether there is similar interest in the Asia Pacific. Second, these studies focused mainly on the progression and review of public health undergraduate education programs (2, 5, 6, 14–16). There are nonetheless a few studies that describe the needs assessment and curriculum development of public health undergraduate education programs. Among the few studies that have done so, these were mainly from the United Kingdom (UK) (17, 18), Netherlands (19), and the USA (20, 21). Recently, the Ashkelon Academic College also shared the development process of the first undergraduate public health baccalaureate program in Israel (22). As public health undergraduate education is gaining popularity in Asia, more such studies would be needed to inform educators, public health professionals, and policymakers in the region. Third, similar to the West, the demand for a more diverse public health workforce has increased in Asia in recent years (23, 24). For example, the shortfall in public health professionals has been estimated to increase from 26,923 in 2017 to 32,592 in 2026 in India (24). Other than having public health undergraduate programs in place, it will be crucial that the graduates from these programs are equipped with sufficient knowledge and skills. As such, the education and training provided to these students would be key to ensuring that they would be able to function effectively after graduation. Studies on the needs assessment and curriculum development of public health undergraduate education programs would thus be useful for other universities that are considering implementing such programs.

Singapore is a city state with a well-developed healthcare system (25). Public health education has traditionally been offered at the postgraduate level as the Master of Public Health (MPH) or Doctor of Philosophy (PhD) by Singapore's first and only tertiary education institution for public health, i.e., the Saw Swee Hock School of Public Health (SSHSPH) at the National University of Singapore (NUS). Undergraduate students in Singapore typically enter the workforce after completion of their first degrees. Thus, in 2013, the School introduced a minor in public health to provide students with an introduction to public health education. The increased uptake in the minor in public health since its inception spurred the School to explore offering more advanced public health education for undergraduates. Concurrent with the global trend of multidisciplinary degrees leading to improved work prospects (26–28), the NUS senior management (academics holding senior leadership positions) actively encouraged the development of second majors comprising half the workload of a first major, to complement preexisting first majors. Typically, in the NUS, a student is required to complete 120 modular credits (MCs) for a bachelor's degree program and 160 MCs for a similar program with honors, with a minimum prescribed cumulative average grade. The bachelor's degree program requires the students to choose a first major offered by the faculty they are enrolled in, and students may pursue the honors program associated with it. All students are required to fulfill both faculty and university requirements. Almost all degree programs allow students to complete elective modules from other faculties, and students interested in areas outside of their first major can choose to fulfill the minor requirements (24 MCs of prescribed modules), which contributes to the breadth of learning envisaged for undergraduate education in the NUS. Students who wish to deepen their interest further may opt for a second major program (48 MCs of prescribed modules). They are, however, not allowed to pursue the honors program with the second major or graduate with a degree in the second major alone, as the second major will only be reflected on the student's transcript. In short, it is compulsory for a student to have a first major, but not a second major or minor (29). The objective of this study was to describe the needs assessment and curriculum development of a second major in public health for undergraduates, the first in Singapore.

## Methods

### Framework Used for Needs Assessment and Curriculum Development

A review of pedagogical literature was conducted using resources from ERIC (Education Resources Information Center) and iSEEK Education. We used the integrated framework for curriculum development in higher education, which consists of five stages (30). In Stage 1, both internal and external environments are assessed on the need for a new curriculum. In Stage 2, competencies to be developed are identified. In Stage 3, the curriculum is designed and developed after triangulating data from Stages 1 and 2. In Stage 4, pedagogical strategies, which are relevant and effective in imparting the knowledge intended in the curriculum, are identified. In Stage 5, implementation, monitoring, and evaluation of the curriculum are carried out. In this manuscript, we have described Stages 1 to 4 as the curriculum is still in the process of implementation at the time of writing. Stages 1–4 were completed between May 2018 and July 2019.

### Stage 1—Environmental Scanning

Externally, we reviewed reports from the Ministry of Health (31) and job portals [Jobstreet Singapore (32), LinkedIn Jobs Singapore (33), Workforce Singapore (34), and Indeed Singapore (35)] to assess the demand for a more diverse public health workforce in Singapore. This was done between September and November 2018 using keywords such as “public health,” “community health,” “health,” and “undergraduate qualification.” A job position was deemed to be related to public health if it satisfied the three predetermined criteria: (i) there was mention of at least one keyword in the job description; (ii) the job did not involve direct patient care (this meant that positions such as doctors, nurses, dietitians, etc. were excluded); and (iii) the job did not specify that they were only looking for those with postgraduate qualifications.

Internally, we tracked the number of students who have declared a minor in public health in the NUS since its debut in 2013. We also conducted (i) a student survey among those taking the minor in public health in the NUS to assess their interest to completing a major in the same discipline, and (ii) a faculty survey to assess the level of support to offer public health as a major. The methodological details of the two surveys will be elaborated on subsequently.

### Stage 2—Graduate Competencies

We conducted an industry stakeholder survey to assess the types of skill sets that public health organizations valued among junior workers (more details below). We also evaluated two sources of data: (i) public health job descriptions using the same job portals in Singapore from Stage 1, and (ii) competencies listed in public health curriculum accreditation frameworks such as the Council on Education for Public Health (CEPH) (36) and the Agency for Public Health Education Accreditation (APHEA) (37).

### Stage 3—Curriculum Development

A multidisciplinary team of educators comprising the SSHSPH's Vice-Dean (Education), faculty members, and managers evaluated the data collected from Stages 1 and 2. We also reviewed the undergraduate public health programs (focusing on major and bachelor's degree) of other universities in the USA, UK, Australia, and Hong Kong (38). After several meetings from November 2018 to July 2019, based on data triangulation and consensus, we developed a formal curriculum for a second major in public health. We compared our new curriculum with that of other universities and mapped the competencies of the core modules with the competencies of the CEPH framework.

Inputs from the Senior Management of SSHSPH were also sought. The curriculum was subsequently presented to the University Committee on Educational Policy (UCEP), a committee of the Senate (the highest level of academic body chaired by the NUS President) in August 2019. The role of the UCEP is to examine and make recommendations concerning educational policies to the Senate. The curriculum was also reviewed by the Board of Undergraduate Studies (BUS), which examined and made recommendations to the Senate on operational matters pertaining to undergraduate education in September 2019. The curriculum was supported by the UCEP and BUS, before it was approved by the Senate in October 2019. This was subsequently endorsed by the Singapore Ministry of Education in December 2019.

### Stage 4—Pedagogical Strategies

We reviewed the pedagogical strategies of the core modules of the curriculum.

## Details of how the Various Surveys Were Conducted

### Student Survey

An anonymous survey was administered via the NUS survey system from May to June 2018. The first part consisted of questions relating to basic demographic data such as age, sex, faculty, and current major. The second part consisted of closed- and open-ended questions on teaching and learning, interest in completing public health as a major, and plans on taking up postgraduate studies.

### Faculty Survey

An anonymous survey was administered online from early to late October 2018. Each existing faculty member or teaching staff who had ever taught in at least one session of a public health minor module was invited to participate. The first part consisted of questions relating to basic demographic data, such as academic designation. The second part consisted of closed- and open-ended questions related to the development of a major program.

### Industry Stakeholder Survey

An anonymous survey was administered online from October to November 2018. Purposive sampling was carried out to ensure that we reached out to a diverse range of stakeholders in the public health sector in Singapore. These stakeholders came from the review of the same job portals in Singapore from Stage 1 and ranged from the public, private, to the non-governmental organizations (NGOs). The first part of the survey consisted of questions relating to basic demographic data such as organization information and professional designation. The second part consisted of closed- and open-ended questions on the development of the major.

## Results

### Stage 1—Environmental Scanning

Our environmental scan revealed that there is a growing demand for public health professionals within the rapidly changing healthcare landscape in Singapore. According to the Healthcare 2020 Masterplan by the Ministry of Health, it was estimated that around 9,000 more people are still needed in public healthcare and community care from 2017 to 2020 (39). Of these, at least 20% of the jobs (1,800 positions) are available as administrative, executive, and managerial positions in public health/healthcare-related statutory boards and agencies, hospital operations and affairs, healthcare human resource and finance, community health, and the regional health systems (39). This coincided with the search on job portals, which revealed close to 1,500 job vacancies related to public health (after accounting for duplicates across the various job portals) (32–35). These positions can be categorized into seven major groups: (i) Analyst, (ii) Clinical Trial Associate/Coordinator, (iii) Executive, (iv) Project or Program Officer, (v) Public Health Officer, (vi) Research Assistant and related jobs, as well as (vii) Workplace, Safety, and Health Officer (32–35).

**Figure 1:**
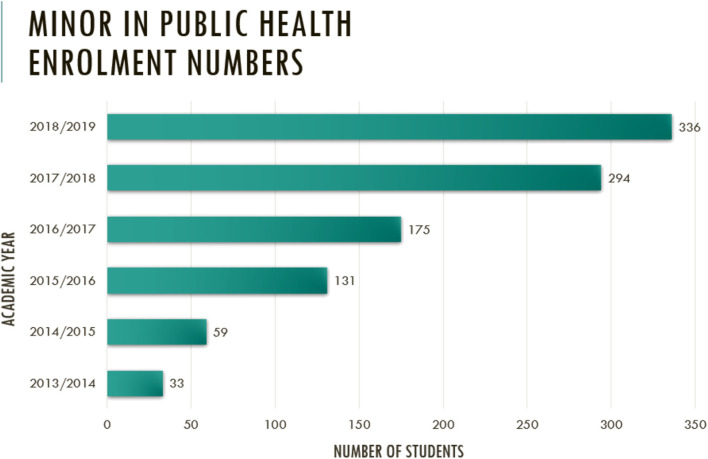
Number enrolled for the minor in public health at the Saw Swee Hock School of Public Health since inception.

There also seemed to be an internal demand for a major in public health. Figure 1 shows the number enrolled for the minor in public health in the NUS. Since its inception, it has become the second most popular minor in the NUS with an average number of 300 students enrolled for the program. Eighty-six out of 298 students participated in the student survey, giving a response rate of about 30%. The demographics of the participants were similar to the demographics of the students who declared a minor in public health. Three out of five respondents were female. Most were from the science faculty (85%), studying life sciences as their first major. The vast majority (88%) indicated an interest to enroll in a second major in public health.

There were five main reasons for studying a second major in public health. Among those who were keen, a quarter felt that public health modules were interesting—“I am passionate about public health and there are many public health modules that I am interested in” (19–25-year-old students studying life sciences as a first major). Another quarter felt that it is relevant in today's context—“I feel that public health is a growing industry in the future as many of the health problems we are facing as a first world country are social in nature. The era of an aging population also adds on to the importance of public health” (19–25-year-old students studying social work as a first major). One-fifth hoped to obtain more comprehensive training in public health—“There are so many modules that are useful to take but most students are only limited to six due to the minor requirements” (19–25-year-old students studying psychology as a first major). Another one-fifth cited the prospects of securing a job in the field of public health or healthcare—“It is relevant for a career in the healthcare sector” (19–25-year-old students studying statistics as a first major). The last one-tenth felt that it would be complementary to their current major—“I also feel it complements my life science degree well as I am interested in healthcare” (19–25-year-old students studying life sciences as a first major).

Twenty-five out of 40 staff participated in the faculty survey. The total response rate was 62.5%. The vast majority expressed strong support for introducing a second major in public health (80%). At the time of the assessment, there was no university in Singapore that offered a major program in public health.

### Stage 2—Graduate Competencies

Thirteen out of 68 stakeholders participated in the stakeholder survey, with a response rate of about 20%. The stakeholders were from various sectors of the public health industry, ranging from health-related governmental agencies/ministries, public hospitals/polyclinics/healthcare facilities, public health–related NGOs, to private organizations. They also held mid to senior level appointments such as Director, Deputy Director, Consultant, Associate Program Officer, and Research Fellow. All respondents expressed a need for staff trained in public health. They expressed a diverse set of public health skills and knowledge that they were seeking among potential junior hires, which were mostly related to fulfilling their organizational or departmental needs. Table 1 shows these desired skill sets, which can be categorized into three main groups: methodological, communication, and thinking skills. The CEPH framework, besides the inclusion of general and foundational public health knowledge and skills, specifically articulates additional soft skills and related competencies (i.e., under Competencies, and Cross-Cutting Concepts and Experiences) important in public health work as part of the accreditation criteria (36). Also as shown in Table 1, the competencies greatly valued by stakeholders were in line with the requirements set out in the CEPH framework, giving further impetus for its use in guiding the development of the second major in public health.

**Table 1:** Desired skill sets of potential junior hires sought by public health industry stakeholders.

**Skill set**	**Component**	**Quotation**	**Domain under the CEPH framework that addressed stakeholder need(s)**
Methodological	Lcate, use, and evaluate public health evidence	“*The staff should possess the skills to do effective literature search, summarize and interpret evidence.” (Director, Abbott)*	Competencies: Ability to locate, use, evaluate, and synthesize public health information
	Interpret basic quantitative and qualitative data	“*We need officers mainly with quantitative method skills.” (Deputy Head, Research Division, National Skin Center)* “*With the changing landscape in public health, we need people to be able to interpret basic qualitative results too.”* *(Research Fellow, Tan Tock Seng Hospital)*	General Curriculum Domain: Basic statistics Foundational Domain: Basic concepts, methods, and tools of public health data collection, use, and analysis and why evidence-based approaches are an essential part of public health practice
	Design a study to collect, analyze, and interpret data	“*To be able to design and conduct a research study on nutrition topics relevant to human health.” (Director, Abbott)*	Cross-Cutting Concepts and Experiences: Research methods Ethical decision-making as related to self and society
Communication	Ability to communicate effectively, both in written and oral forms	“*To be able to communicate to stakeholders and the public.” (Senior Consultant, Ministry of Health)* *“The staff should be able to communicate effectively in writing and exhibit clear communication skills.”* *(Head of General Staff, Singapore Armed Forces)*	Competencies: Ability to communicate public health information, in both oral and written forms, through a variety of media and to diverse audiences Foundational Domain: Basic concepts of public health-specific communication, including technical and professional writing and the use of mass media and electronic technology
	Collaborative skills/working in teams	“*Globally, our organization has a great need for field workers and office staff who can work well in teams. As an international medical humanitarian organization in over 70 countries, we are always in need of professionals who can function effectively in teams.”* *(Research Officer, Medecins Sans Frontieres)*	Cross-Cutting Concepts and Experiences: Teamwork and leadership Professionalism
Thinking	Systems thinking	“*We need officers who are able to apply systems thinking when tackling the modern public health issues. For example, we are designing a healthy precinct framework which advocates the coordinated implementation of a comprehensive set of health promoting interventions, together with active participation of the community, we would need the staff to be able to apply the socio-ecological framework for health promotion, incorporating behavioral nudges, attitudinal changes, community activation and engagement, environmental modification, and health in all policies.”* *(Director, Ministry of Health)*	Foundational domains: Concepts of population health, and the basic processes, approaches, and interventions that identify and address the major health-related needs and concerns of populations Fundamental concepts and features of project implementation, including planning, assessment, and evaluation The underlying science of human health and disease, including opportunities for promoting and protecting health across the life course The socioeconomic, behavioral, biological, environmental, and other factors that impact human health and contribute to health disparities Cross-Cutting Concepts and Experiences: Systems thinking Advocacy for protection and promotion of the public's health at all levels of society
	Cross-disciplinary and critical thinking	“*There is a need to have officers who can think beyond the scope of traditional public health and to apply the knowledge or skills they might have learnt from other disciplines since public health is becoming more multi-disciplinary. These officers should also be able to think critically considering that public health issues will be increasingly complex in the future.”* * (Director, Vital Strategies/International Union Against Tuberculosis and Lung Disease)*	Cross-Cutting Concepts and Experiences: Critical thinking and creativity Systems thinking

Based on the review of job descriptions of public health roles from the portals, we found that many of the job postings specified requests for graduates from diverse backgrounds, e.g., major in life sciences, psychology, sociology, business, economics, or statistics. While these roles also required a complementary, albeit heterogeneous, mix of public health skills and knowledge, common skill sets were described across all job categories, particularly soft skills such as verbal and written communication skills, and the ability to work effectively both in teams and independently. These findings were congruent with the results of the stakeholder survey, supporting the timely and strategic development of a second major in public health in preparing undergraduates for a career in the field and in meeting the demands of the workforce.

### Stage 3—Curriculum Development

Table 2 shows the overall structure of the second major curriculum to be offered to all students of the university from Year 2 onwards. The aims of the program are to (i) prepare students for career opportunities in organizations engaged in public health work, (ii) equip students with the foundational knowledge, skills, and approaches needed to characterize and address today's public health challenges in Singapore, Asia, and the world, and (iii) develop students' perspectives and soft skills for working in public health teams. Students have to take a total of 12 modules, of which 6 are core and 6 are elective. The capstone module is a 320-our internship module where students will be attached to public health–related agencies, organizations, or NGOs for work experience.

**Table 2:** Overall curriculum structure of public health second major offered by the Saw Swee Hock School of Public Health, National University of Singapore.

**Overall structure**	**6 core modules + 6 elective modules = 12 modules (48 modular credits, MCs)**
Core modules	**24 MCs (6 Modules)** 1. SPH2001 fundamental public health methods 2. SPH2002 public health and epidemiology 3. SPH2003 systems and policies to improve health 4. SPH2004 lifestyle, behavior and public health 5. SPH2005 health, society and the social determinants 6. SPH3001 public health practice (capstone module)
Elective modules	**24 MCs (6 Modules)** • At least 12 MCs at level 3,000 or above • At least 12 MCs of SPHXXXX modules (maximum 4 MC of Level 5,000 modules) • Maximum 4 MC from the quantitative and qualitative methods modules **SPH modules (meaning modules offered by the School of Public Health)** **Level 2000 modules** • SPH2202 public health nutrition • SPH2401 introduction to global health • SPH2402 health in the later years • SPH2801 health of the poor in Asia **Level 3000 modules** • SPH3101 biostatistics for public health • SPH3202 infectious disease epidemiology and public health • SPH3203 prevention and control of non-communicable diseases • SPH3401 designing public health programs • SPH3403 public health economics • SPH3404 physical activity for better population health • SPH3501 introduction to public health communication **Level 5000 modules** **(Pre-requisites: All the core modules except the capstone module)** • SPH5003 health behavior and communication • SPH5004 introduction to health policy and policy analysis • SPH5204 nutrition and health—fundamentals and applications • SPH5401 health economics and financing • SPH5402 management of healthcare organizations • SPH5403 medical and humanitarian emergencies • SPH5406 contemporary global health issues **Modules offered by other faculties, schools, and departments** **Level 2000 modules** • GE2206 geographies of life and death • GE2227 cartography and visualization • SC2211 medical sociology • ENV2103 environment and public health **Level 3000 modules** • FST3202 nutrition and disease prevention • LSM3224 molecular basis of human diseases • LSM3225 molecular microbiology in human diseases • PL3242 health psychology • PR3202 community health and preventative care • SW3207 social work in medical settings • SW3217 mental health and illness • NM3237Y health communication **Level 4000 module** • LSM4217 functional aging
	**Quantitative and Qualitative methods modules** **Level 1000 modules** • ST1131 introduction to statistics • ST1232 statistics for life sciences **Level 2000 modules** • EC2303 foundations for econometrics • GE2101 methods and practices in geography • NM2103 quantitative research methods • PL2131 research and statistical methods I • SC2101 methods of social research • DAO2702 programming for business analytics • RE2801/ RE3201 research methodology in real estate • BN2102 bioengineering data analysis • ST2132 mathematical statistics • ST2334 probability and statistics • UQF2101G quantitative reasoning foundation: quantifying nuclear risks • UQF2101I quantitative reasoning foundation: quantifying environmental quality **Level 3000 modules** • PS3257 political inquiry • SW3101 social work research methods • PF3105 research methods • CN3421 process modeling and numerical simulation • MA3259 mathematical methods in genomics **Level 4000 module** • PS4314 data analytics in political science **Level 5000 module** • IE5205 healthcare system and analytics

The curriculum map of the core modules of the second major in relation to the respective competencies of the CEPH is shown in Table 3. The six core modules were designed to fulfill the foundational requirements and competencies under the CEPH framework, and the skills desired by prospective employers in entry-level positions as highlighted in the stakeholder survey.

**Table 3:** Curriculum mapping of the core modules of the second major to the respective competencies of the Council on Education for Public Health.

**Domain**	**Domain-specific requirements (CEPH)**	**SPH2001 fundamental public health methods**	**SPH2002 public health and epidemiology**	**SPH2003 systems and policies to improve health**	**SPH2004 lifestyle, behavior, and public health**	**SPH2005 health, society, and the social determinants**	**SPH3001 public health practice (Capstone module)**
General curriculum domain	The foundations of scientific knowledge, including the biological and life sciences and the concepts of health and disease	–	X	–	X	–	–
	The foundations of social and behavioral sciences	–	–	X	X	X	–
	Basic statistics	X	–	–	–	–	–
	The humanities/fine arts	–	–	–	X	X	–
Foundational domain	Basic concepts, methods, and tools of public health data collection, use, and analysis and why evidence-based approaches are an essential part of public health practice	X	X	–	–	–	–
	Concepts of population health, and the basic processes, approaches, and interventions that identify and address the major health-related needs and concerns of populations	–	X	X	X	X	–
	Underlying science of human health and disease, including opportunities for promoting and protecting health across the life course	–	X	X	X	–	–
	Socioeconomic, behavioral, biological, environmental, and other factors that impact human health and contribute to health disparities	–	–	–	X	X	–
	The history and philosophy of public health as well as its core values, concepts, and functions across the globe and in society	–	X	X	–	–	–
	Fundamental concepts and features of project implementation, including planning, assessment, and evaluation	–	X	–	X	–	X
	Fundamental characteristics and organizational structures of the Singapore health system as well as the differences between systems in other countries	–	–	X	–	–	X
	Basic concepts of legal, ethical, economic, and regulatory dimensions of health care and public health policy and the roles, influences, and responsibilities of the different agencies and branches of government	X	–	X	–	–	X
	Basic concepts of public health-specific communication, including technical and professional writing and the use of mass media and electronic technology	X	X	–	X	X	X
Competencies	Ability to communicate public health information, in both oral and written forms, through a variety of media and to diverse audiences	X	X	–	X	X	X
	Ability to locate, use, evaluate, and synthesize public health information	X	X	X	X	–	X
Cumulative and experiential activities	Cumulative, integrative, and scholarly or applied experience or inquiry project that serves as a capstone to the education experience. These experiences may include, but are not limited to, internships, service-learning projects, senior seminars, portfolio projects, research papers, or honors theses	X	–	X	–	X	X
Cross-cutting concepts and experiences	Advocacy for protection and promotion of the public's health at all levels of society	–	–	X	X	X	X
	Community dynamics	–	–	X	–	X	X
	Critical thinking and creativity	–	X	X	X	X	X
	Cultural contexts in which public health professionals work	–	X	X	X	–	X
	Ethical decision-making as related to self and society	X	X	X	X	X	X
	Independent work and a personal work ethic	X	X	X	X	X	X
	Networking	–	–	–	X	–	X
	Organizational dynamics	–	X	X	X	X	X
	Professionalism	X	X	–	X	–	X
	Research methods	X	X	–	X	–	–
	Systems thinking	–	X	X	X	–	X
	Teamwork and leadership	X	X	X	X	X	X

The first core module, SPH2001 Fundamental Public Health Methods, provides students with an overview of research methods, and quantitative and qualitative approaches commonly used in public health research and practice. It aims to equip students with the foundational knowledge and skills in public health research and practice such as introductory statistics, locating and evaluating public health information, interpreting basic quantitative and qualitative public health data, as well as communicating public health information in both oral and written forms.

The second core module, SPH2002 Public Health and Epidemiology, covers key concepts in epidemiology, including measurement of disease burden, evaluation of risk factors for diseases of public health importance, evaluation of interventions like new vaccines and therapies, and critical appraisal of research evidence to inform public health policy.

The third core module, SPH2003 Systems and Policies to Improve Health, provides students with an introductory overview of health systems and policies that have the ability to shape an individual's and the population's well-being. The module uses Singapore's healthcare system as a case study to explain the organization of health systems and the policy responses to public health challenges that arise within the context of these systems.

The fourth core module, SPH2004 Lifestyle, Behavior and Public Health, introduces principles of behavioral change and health promotion and how they apply to behavioral lifestyle factors and disease prevention. It provides an overview of important behavioral lifestyle factors (i.e., tobacco consumption, dietary behavior, physical activity, sedentary behavior, alcohol consumption, sexual behavior, and sleeping behavior) and their impact on individual and population health. Examples of past and present public health approaches to target these lifestyle factors are discussed. Students also learn to consider effectiveness and ethics of health promotion strategies in the context of discussed lifestyle factors.

The fifth core module, SPH2005 Health, Society and the Social Determinants, explores contemporary technological, ethical, political, and cultural debates in health, healing, and well-being. It adopts a multidisciplinary approach in examining the different understandings of “health” in society, via thinking critically about real-world health issues and their management. Drawing from disciplines such as medical anthropology, urban sociology, and human geography, students investigate how “health” implicates—and is implicated by—the lives of individuals and societies.

The last core module, SPH3001 Public Health Practice, is a capstone that introduces students to the public health infrastructure and functions in Singapore, as well as provides hands-on exposure to work by way of attachments at public health–related agencies, organizations, or NGOs. It allows students to explore career opportunities in public health, develop related essential skills, specifically soft skills such as management of resources, time, money, and human, interpersonal relationships, communication, and advocacy, and provides practical exposure to selected public health careers.

The electives have been structured to allow flexibility for students to either broaden their perspectives or deepen their understandings in a specific area of focus. In addition to the elective modules offered by the School, modules of public health relevance from the Faculty of Arts and Social Sciences, Faculty of Engineering, Faculty of Science, School of Business, and the School of Design and Environment can contribute toward the fulfillment of the second major requirements.

Our curriculum is generally aligned with undergraduate public health programs in other universities such as the George Washington University, Johns Hopkins University, and the Indiana University Bloomington School of Public Health in the USA, Birmingham University and University of Brighton in UK, Monash University and University of New South Wales in Australia, and the Chinese University of Hong Kong (38). Of the programs reviewed, the core modules of our major align most closely with the USA-based programs, which include basics in epidemiology, a capstone, and social/behavioral modules in their core offerings (38).

### Stage 4—Pedagogical Strategies

The core modules are designed to ensure that students would have a common set of skills and approaches to maximize their employability in the diverse landscape of public health. Diverse pedagogical strategies are utilized. Table 4 provides an overview of the various pedagogical strategies used in the core modules of the curriculum. These include lectures, tutorial/breakout sessions, presentations/interactive discussions, reports/reflective essays/briefs, field visits/field work, simulation exercises, as well as activities involving case-based learning, collaborative learning, project-based learning, and service-based learning.

**Table 4:** Pedagogical strategies employed for each core module of the public health second major.

**Core module**	**Pedagogical strategies and approaches**									
	**Lecture/seminar**	**Tutorial/break out session**	**Presentation/interactive discussion**	**Report/reflective essay/brief**	**Field visit/field work**	**Simulation exercise**	**Case-based learning**	**Collaborative learning**	**Project-based learning**	**Service-based learning**
SPH2001 fundamental public health methods	✓	✓	✓	✓	–	–	–	✓	–	–
SPH2002 public health and epidemiology	✓	✓	✓	✓	–	✓	–	✓	–	–
SPH2003 systems and policies to improve health	✓	✓	✓	–	✓	–	–	✓	–	–
SPH2004 lifestyle, behavior and public health	✓	✓	✓	–	–	–	✓	✓	–	–
SPH2005 health, society and the social determinants	✓	✓	✓	✓	✓	–	✓	✓	–	–
SPH3001 public health practice (capstone module)	✓	✓	✓	✓	–	–	–	–	✓	✓

## Discussion

Undergraduate public health education is still a new concept in Singapore. Curriculum development is both a science and an art (a science because it has to be informed by scholarship and theoretical models, plus empirical research; and an art because it needs to fulfill the need and wants of various stakeholders in the dynamic public health landscape). In this paper, we have described how we have used an integrated and evidence-based approach to assess the needs and develop the curriculum of the first public health major in Singapore. We have also highlighted how we have involved all relevant stakeholders, i.e., students, faculty, and industry players, in this process by ensuring inclusiveness and incorporating their views in curriculum development.

There are a few reasons why this study, and the subsequent adoption and implementation of the second major in public health, is of significance. First, as we are pioneering the development of the first major in public health in Singapore, it is important to have a window of opportunity for us to achieve this goal. Fortunately, the time was ripe for this to occur. Not only were there employment opportunities in the public health sector and an internal demand within the university, the environment was also conducive enough to allow for changes to the undergraduate public health education amidst the background of a growing national public health network in Singapore. This was also in line with our underlying educational philosophy, which sought to build an educated citizenry through a well-trained workforce in public health so that they can contribute to the society by fostering healthier communities in Singapore and the region. Moreover, the School will be leveraging on the support of faculty members who have been teaching in the existing minor for the new program. In addition, the School has been recruiting new faculty members and this includes new hires with health promotion, behavioral change, and public health communication expertise. Therefore, the School does not anticipate significantly greater demands on manpower or teaching facilities that will compromise the quality of the new program.

Second, our list of electives has included modules from other faculties and schools, which have relevance to the public health curriculum. This is distinct to our program among other major programs at the NUS. The recognition of courses from other disciplines as part of our second major curriculum contributes to the richness of the interdisciplinary nature of public health as a field, and enriches the curriculum through the provision of opportunities to develop cross-disciplinary perspectives. Given that modern public health practice often requires professionals from different disciplines to work together, it is imperative to embrace diversity in our curriculum. This has been among the philosophies that were prioritized throughout the journey of developing a new curriculum to equip more public health professionals with the skill sets required in the complex public health landscape. As public health has evolved from a narrow, disease-focused discipline to a broader multidisciplinary and multisectoral endeavor, paradigms from other disciplines have been increasingly utilized to bring innovations in public health undergraduate education (40, 41). The diversity of students that we envision who will enroll in this second major is thus a foreseen strength of the program, given the opportunities for an appreciation of a broad range of disciplines, as well as windows to encourage interdisciplinary learning. The increasingly interconnected nature of issues faced by the future public health worker often means that various tools and techniques are needed, which have to come from exposure to various disciplines as early as during the undergraduate years. This innovative form of learning is believed to provide fertile ground for the cultivation of future healthcare leaders, system thinkers, and innovators rather than routine thinkers for the field (40, 41).

Third, in view of the diversity of students who will be expected to enroll in the second major, scaffolding—the use of a variety of instructional techniques to move students progressively toward stronger understanding and, ultimately, greater independence in the learning process (42)—is hence essential. Students are encouraged to complete the core modules (except the capstone module) before embarking on the electives. Two core modules, SPH2001 Fundamental Public Health Methods and SPH2002 Public Health and Epidemiology, were designed to provide the foundational approaches and concepts to public health, which are essential and applicable across the diverse spectrum of public health careers, thereby forming the first tier of learning. The second tier comprises the rest of the core and elective modules that not only build upon the foundational concepts and skills from the first tier modules, but also equip students with skills and approaches that may be more specialized (e.g., public health economics, program design, health systems, and policies) or contextual (e.g., modules focused on communicable/non-communicable diseases, nutrition, physical activity, aging) in nature. The final tier of learning entails the capstone internship module, which aims to expose students to public health career opportunities and enable them to apply the skills acquired from the program cumulatively. Through this capstone module, students get the opportunity to be immersed in an actual workplace before they formally enter the workforce. In fact, internship experiences have been consistently emphasized in other undergraduate public health programs abroad (38). This integral feature in our second major in public health provides students with a strong head start in professional life through experiential learning, a practical approach to education where benefits such as increased knowledge, sharper skills, increased confidence, and greater cultural sensitivity have been reported, in addition to well-sought-after opportunities for graduate programs and jobs (43–45).

## Future Directions

At the time of writing, we are at Stage 5 of the integrated framework for curriculum development where implementation, monitoring, and evaluation are still being carried out. After the curriculum is launched in January 2021, we will periodically assess the attainment of the program's learning outcomes through indirect (e.g., student, alumni, faculty, and industry stakeholder surveys) or direct evaluation and feedback pathways (e.g., assessment tasks in the various modules). Student learning and success will be evaluated based on performance in modular assessments via NUS-regulated assessment and tracking methods. In addition, student perceptions of learning and helpfulness of the program to career development and advancement will be obtained from annual school-level surveys. Student feedback on teaching effectiveness for all modules will also be gathered at the end of each semester. The data will be disseminated to module coordinators and the School's education office for review and action on a regular basis, to ensure the continuous alignment of teaching and learning to program outcomes, as well as to maintain baseline quality checks on pedagogy and assessment.

## Conclusion

While articles featuring detailed narratives of new and innovative public health curricula are often published, the needs assessment and the curriculum development processes are rarely explained. In this paper, we have described the rationale, underlying philosophies, as well as the needs assessment and curriculum development of a major in public health in Singapore using an integrated and evidence-based approach. We hope that the lessons that we have learned from this journey will serve to inform other universities in the Asia Pacific region that are considering implementing such programs and broadening their offerings in public health education.

## Data Availability Statement

The datasets analyzed for this study were included in the article.

## Ethics Statement

This was an exempt review study according to the guidelines of the National University of Singapore Institutional Review Board. Written informed consent for participation was not required for this study in accordance with the national legislation and the institutional requirements.

## Author Contributions

All authors listed have made a substantial, direct, and intellectual contribution to the work, and approved it for publication.

## Conflict of Interest

The authors declare that the research was conducted in the absence of any commercial or financial relationships that could be construed as a potential conflict of interest.
